# Preliminary evaluation of the proteomic profiling in the hippocampus of aged grazing cattle

**DOI:** 10.3389/fnagi.2023.1274073

**Published:** 2023-10-27

**Authors:** Flora Cozzolino, Luisa Canè, Luigi Sacchettino, Maria Claudia Gatto, Ilaria Iacobucci, Claudia Gatta, Davide De Biase, Evaristo Di Napoli, Orlando Paciello, Luigi Avallone, Maria Monti, Danila d’Angelo, Francesco Napolitano

**Affiliations:** ^1^CEINGE-Biotecnologie Avanzate “Franco Salvatore”-Via G. Salvatore, Naples, Italy; ^2^Department of Chemical Sciences, University of Naples, Federico II, Naples, Italy; ^3^Department of Translational Medical Sciences, University of Naples Federico II, Naples, Italy; ^4^Department of Veterinary Medicine and Animal Production, University of Naples Federico II, Naples, Italy; ^5^Department of Pharmacy, University of Salerno, Fisciano, Italy

**Keywords:** aging, bovine, brain, hippocampus, proteomics

## Abstract

Brain aging is a physiological process associated with physical and cognitive decline; however, in both humans and animals, it can be regarded as a risk factor for neurodegenerative disorders, such as Alzheimer’s disease. Among several brain regions, hippocampus appears to be more susceptible to detrimental effects of aging. Hippocampus belongs to limbic system and is mainly involved in declarative memories and context-dependent spatial-learning, whose integrity is compromised in an age-dependent manner. In the present work, taking advantage of liquid chromatography–tandem mass spectrometry (LC–MS/MS)-based proteomics, we sought to identify proteins differentially expressed in the hippocampus of the aged grazing milk cows. Our exploratory findings showed that, out of 707 identified proteins, 112 were significantly altered in old cattle, when compared to the adult controls, and functional clusterization highlighted their involvement in myelination, synaptic vesicle, metabolism, and calcium-related biological pathways. Overall, our preliminary data pave the way for the future studies, aimed at better characterizing the role of such a subcortical brain region in the age-dependent cognitive decline, as well as identifying early aging markers to improve animal welfare and husbandry practices of dairy cattle from intensive livestock.

## 1. Introduction

Brain aging is a physiologic process, characterized by molecular, structural, and behavioral changes that, eventually bring about cognitive dysfunctions. In this line, progressive deficits in learning and memory occur at different extent and brain regions, like hippocampus, which is particularly vulnerable to aging process in mammals, including domestic and laboratory animals, as well as humans (Peters, 2006; Youssef et al., 2016). Pioneering studies from O’Keef and colleagues highlighted the structural-functional relationship for such a subcortical region, so it can be regarded as a key player for spatial navigation and memory consolidation (O'Keefe and Dostrovsky, 1971). Although precise mechanisms underlying age-dependent decay of cognitive abilities need to be still deeper addressed, preclinical and clinical studies documented a correlation between age-dependent cognitive decline and decrease of hippocampal volume (Driscoll et al., 2006). Alongside structural changes occurring over time, aging has the potential to severely modify the physiology of the synaptic strength and plasticity within the hippocampus. Synaptic plasticity relies on the ability of neurons to make and break connections, thereby modifying the efficacy of the preexisting ones, that impact on subsequent thoughts, feelings, and behavior (Citri and Malenka, 2008). Long-Term Potentiation (LTP) and Long-Term Depression (LTD) represent the neurobiological substrate of the hippocampal synaptic plasticity which, overall, appear impaired during aging, and dysfunctional in neurological disorders. In this respect, studies in rodents showed a rapid age-dependent decay of hippocampal LTP, since old rats had poorer performance in spatial memory abilities, together with a greater hippocampal depotentiation and lower threshold for LTD onset (Norris et al., 1996; Errico et al., 2008; Lister and Barnes, 2009; Errico et al., 2011a,b). The age-dependent reduction of hippocampal synapses is associated with morphological changes of dendritic spines and dendrites, in addition to alterations of synaptic vesicle protein levels, which are accountable for neurotransmitter release during the physiological activity of the hippocampus and, in turn, contribute to cognitive decline (Wang et al., 2007; Leal and Yassa, 2015; Truckenbrodt et al., 2018). Among the brain aging complexity culprits, oxidative stress, glucocorticoid-related dysfunctions, epigenetic and nutritional factors might play a prominent role in the hippocampal physiology (Bettio et al., 2017). In this framework, some of the major traits of cognitive decline might be related to the pathological extracellular Aβ deposition and intracellular tau aggregation, together with an impaired intracellular degradative pathway (autophagy), which are regarded as hallmarks for the early onset of Alzheimer’s disease in humans and animals (Reddy et al., 2018; Rao et al., 2022). Accordingly, the occurrence of AD-like pathology was reported in some aged cow, since they showed scattered Aβ deposits and substantial amount of amyloid accumulation in cortex and hippocampus (Moreno-Gonzalez et al., 2021). Thus, considering the crucial role for the hippocampus upon molecular and behavioral symptoms during aging, a deeper characterization of the age-dependent brain physiology paves the way for the development of strategies aimed at counteracting, or slowing down, the detrimental effects of the hippocampal decline in humans and farm animals as well. Therefore, in the present explorative study, taking advantage of liquid chromatography–tandem mass spectrometry (LC–MS/MS)-based proteomics, we sought to identify proteins differentially expressed in the hippocampus of the aged grazing milk cows, to shed light on the biological processes mainly impaired during aging, and setting the ground to increase animal welfare and husbandry practices.

## 2. Methods

### 2.1. Animals

For the present study, two groups of animals were employed: an adult group of 7–12 years (*n* = 4) and aged group of 16–24 years (*n* = 5). Samples were collected post-mortem in an abattoir in Campania Region, Italy, performing inspection to ensure the absence of any apparent clinical illness or neurological signs (gait abnormalities, weakness, and decreased mental status).

### 2.2. Ethical statement

Even though our research includes animal tissues, the study did not require consent or ethical approval according to European Directive 2010/63/EU because all sampling procedures from animals were performed during post-mortem inspection. However, the animals were slaughtered rigorously in line with European regulations (CE no: 1099/2009 of 24 September 2009) that assure the protection and welfare of animals at the time of killing. The owner of the abattoir and the veterinary inspector responsible for the sanitary surveillance granted permission to collect the samples.

### 2.3. Proteomics analysis

Hippocampus samples from 4 adult and 5 aged dairy cattle were treated as reported in Cozzolino et al. (2021) to carry out a label free shotgun differential proteomics analysis (Andolfo et al., 2023). Peptide mixtures were dissolved in 100 μL of formic acid 0.2% and 2 μL were analyzed on an LTQ Orbitrap XL (Thermo Fisher Scientific) coupled to the nanoACQUITY UPLC system (Waters; Bertini et al., 2021). The software MaxQuant (v. 1.5.2.8) was employed for protein identification and quantification through raw files using the following parameters: UniProt Database; Taxonomy: *Bos taurus* (Bovine); enzyme: trypsin; 1 missed cleavages allowed; fixed changes: carbamidomethylation (C); variable changes: oxidation (M); Gln-pyro-Glu (N-Term Q); minimum number of peptides for identification: four with at least two unique; minimum number of peptides for quantification four; FDR: 0.01; peptide tolerance: 20 ppm (Iacobucci et al., 2022). Data are available via ProteomeXchange with identifier PXD044404. For the phosphorylation analysis, Phospho(STY) was set as variable modification and the generated Phosphosites(STY) file was used for the statistical analysis with Perseus software.

### 2.4. Functional enrichment analyses

Differentially expressed proteins were statistically filtered as described below, and those showing |log2FCs| ≥ 0.5 were collected in a unique list, which was analyzed using the ClueGO + CluPEDIA extension app of Cytoscape (v3.9.1). The biological processes database of Gene Ontology (GO) *Bos Taurus* was queried by applying the Benjamini-Hochberg correction with a value of *p* < 0.05 (Palinski et al., 2021).

### 2.5. Multiple reaction monitoring analyses

Multiple Reaction Monitoring (MRM) analysis was carried out for the validation of some putative targets of each functional network. By using Skyline v20.1.0.155 (MacCoss Lab Software, Dept of Genome Sciences, UW), for each protein, at least three prototypic peptides and three transitions for each deriving parent ion were selected and monitored by using a Xevo-TQS triple-quad mass spectrometer, coupled to a nanoAcquity UHPLC (Waters, Milford, MA, United States) equipped with IonKey CHIP interface, according to Cozzolino et al. (2020). Each run was analyzed in duplicate and the total area of each peptide transition was used for the relative quantification of the specific protein, by using the area of actin peptide ions transitions for normalization (Berkman et al., 2019).

### 2.6. Statistical analysis

The Perseus Software (1.6.15.0) was used for statistical analysis of differentially expressed proteins, by analyzing LFQ Intensity values, according to normalization and imputation criteria reported in (Andolfo et al., 2023). Proteins with more than 70% of non-valid values (zeros) were removed, and the data were normalized by applying a logarithmic scale [log_2_(LFQ)]. Additionally, Perseus imputation was used to allow the replacement of invalid values with numerical values (downshift of the standard deviation of 1.8 and a width of 0.3). The statistical significance of the difference in protein expression was calculated using Student’s T-test. On the other hand, the phosphorylation analysis was performed by filtering entries with more than 50% of non-valid values, and the missing values were replaced by imputation as explained above. The statistical test was performed using a Student’s T-test with a Benjamini-Hochberg corrected value of *p* threshold of 0.05 (Supplementary Table S1). The distribution of the samples was defined by using the platform Principal Component Analysis (PCA) of Perseus software. For MRM experiments, a t-test was performed to define the statistical significance of measured FCs of selected proteins as aged vs. adult, by using GraphPad Prism 9.

## 3. Results

### 3.1. Proteomics profiling of hippocampus from aged grazing cattle

**Figure 1:**
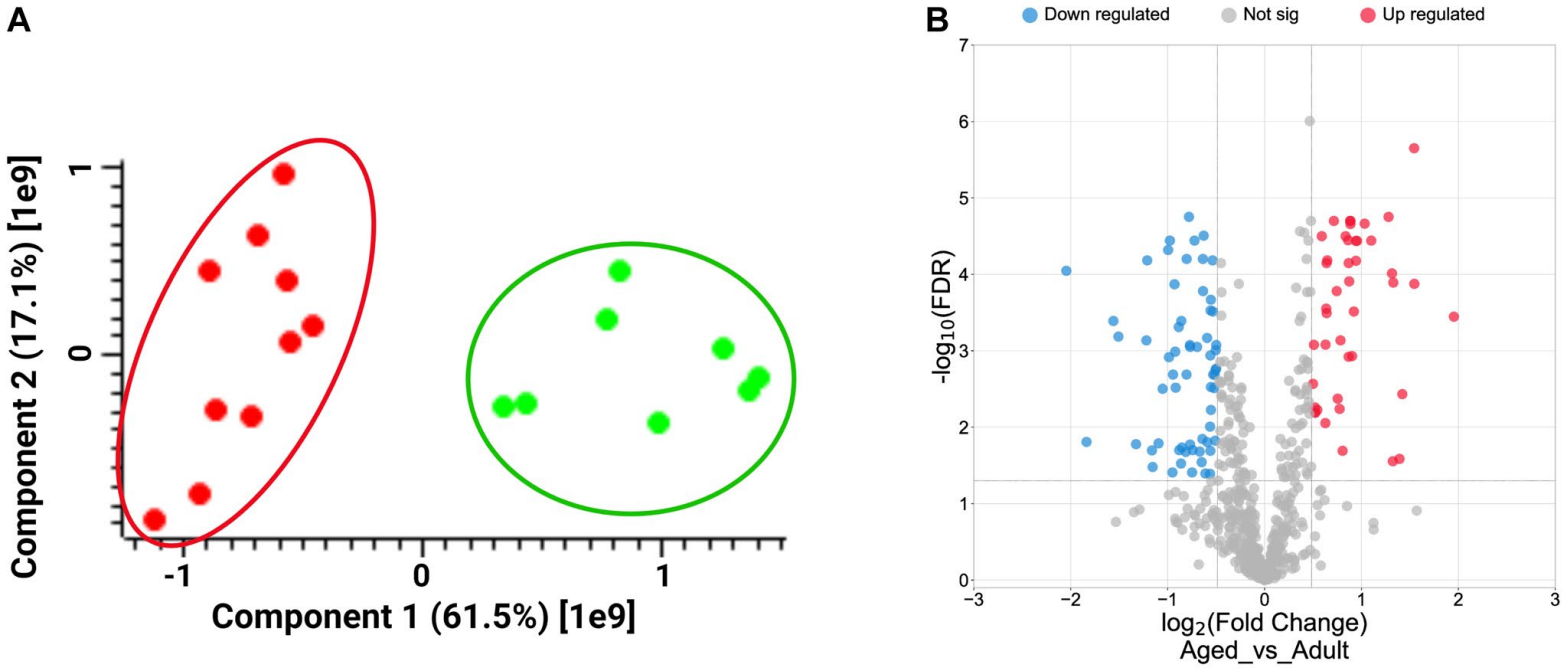
Panel **A**: Plot of Principal Component Analysis (PCA) displays the distribution of biological samples from adult (green dots) and aged (red dots) hippocampus brain section according to Perseus analysis. Panel **B**: Volcano plot showing the distribution of up- (red dots, log_2_FCs ≥ 0.5) and down-regulated proteins (blue dots, log_2_FCs ≤ −0.5), according to their Log_2_ FCs and -Log_10_FDR values.

A shotgun label free proteomics approach was carried out to compare the hippocampus’s profiling from 5 aged (16–24 years old) grazing cattle to that deriving from 4 adult specimens (7–12 years old). Tissue samples were treated as described in the methods section, and protein extracts digested by trypsin according to a classical shotgun protocol. Peptide mixtures were analyzed by LC–MS/MS and protein identification and quantification according LFQ intensity values were achieved by using MaxQuant software, as reported in Supplementary Table S1. Principal Component Analysis was carried out by using Perseus, and the results, showed in the PCA plot (Figure 1A), highlight the perfect agreement among samples belonging to the same set, suggesting a high level of homogeneity from a biological point of view. Conversely, the distance between the two sets of samples, adult and aged, is in line with their biological differences.

A t-test was then carried out to extract the statistically significant proteins from the starting protein groups. We selected 221 proteins showing FDR values<0.05, and the Fold Changes (FCs) were calculated by averaging the LFQ values between the two replicates within a same sample set. Finally, we obtained 62 down- (log_2_FCs ≤ −0.5) and 43 up-regulated proteins (log_2_FCs ≥ 0.5; Supplementary Table S1) as graphically reported in the volcano plot in Figure 1B.

A larger number of down-regulated proteins in comparison to the up-regulated suggested a prevalent loss of functions in the aged brains.

### 3.2. Functional enrichment analyses

**Figure 2:**
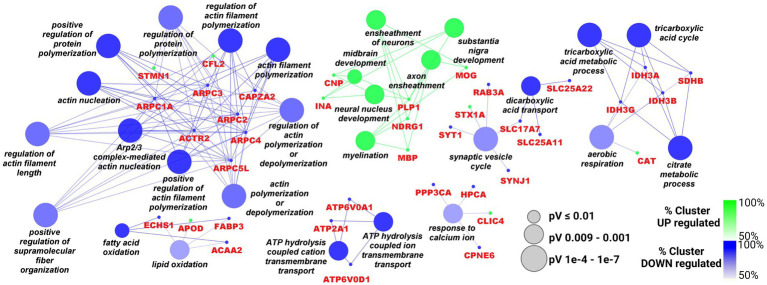
Cytoscape graphical representation of Biological Processes including up- (green) and down-regulated (blue) proteins. The GO database was used; a Benjamini-Hochberg correction (value of *p* ≤ 0.05) was applied. Node sizes were correlated with the specific *p* value.

Functional clusterization according to Biological Processes of the 43 up- and the 62 down-regulated proteins was performed by Cytoscape software, with the ClueGO app + CluPEDIA extension and Benjamini-Hochberg as the statistical option, with a cutoff of FDR ≤ 0.05. The resulting Cytoscape networks are reported in Figure 2.

As showed in the Figure 2, the only up-regulated process overcoming the statistical filter was *“axon ensheathment/myelination”* (the green cluster) including CNP, INA, PLP1, MOG, NDRG1, MBP. Among the down-regulated processes, the largest number of proteins were distributed between *“Arp2/3 complex/regulation of actin polimerisation”* (ARPC1A, ARPC2, ARPC3, ACTR2, ARPC4, ARPC5L, CAPZA2, STMN1, CFL2) and *“citrate metabolicprocess/aerobic respiration”* (several Isocitrate Dehydrogenase subunits such as IDH3A, IDH3B, IDH3G and succinate dehydrogenase SDHB are included). Cytoskeleton remodeling might be also associated with another important process, namely “*synaptic vesicle cycle*” (SYT1, STX1A, RAB3A, SYNJ1, SLC17A7), since both can affect the transport and the release of neurotransmitters at different levels. Also, calcium homeostasis, a crucial process for brain functionality, looked impaired in this picture, with the down regulation of PPP3CA, HPCA and CPNE6. Moreover, the cluster *“ATP hydrolysis coupled cation transmembrane transport,”* comprising some subunits of the V0 complex of vacuolar(H+)-ATPase (V-ATPase; ATP6V0D1 and ATP6V0A1), and ATP2A1 can also be associated with cation exchange, including Ca^2+^ ions. However, V-ATPase complex is also responsible for the acidification of various organelles, including synaptic vesicles, and plays an essential role in neuronal development in terms of integrity and connectivity of neurons, through its action on compartment acidification.

### 3.3. Protein fold changes validation

At least one protein belonging to the main functional clusters was selected for validation experiments based on a LC–MSMS approach, known as Multiple Reaction Monitoring (MRM), alternative to the western blot assays. MRM experiments were set up to monitor specific transitions of proteotypic peptides selected for each protein target, by using Skyline software. A transition consists in a couple of m/z values including the parent ion and a daughter ion generated upon fragmentation procedure. For each peptide more than one transitions can be foreseen, according to its amino acid sequence. Tryptic mixtures from all adult and aged samples were analyzed in MRM mode and two or more transitions of at least three proteotypic peptides were monitored for each protein. Each sample was run in duplicate and the average areas, normalized with the areas measured for ACTIN-derived transitions in the same sample, were statistically filtered and employed for calculating the FCs of the specific protein in aged vs. adult. Supplementary Table S2 reported the proteins validated with their relative peptides, transitions, MRM data, and the FCs measured in the targeted approach (MRM) in comparison with the untargeted method (label free).

**Figure 3:**
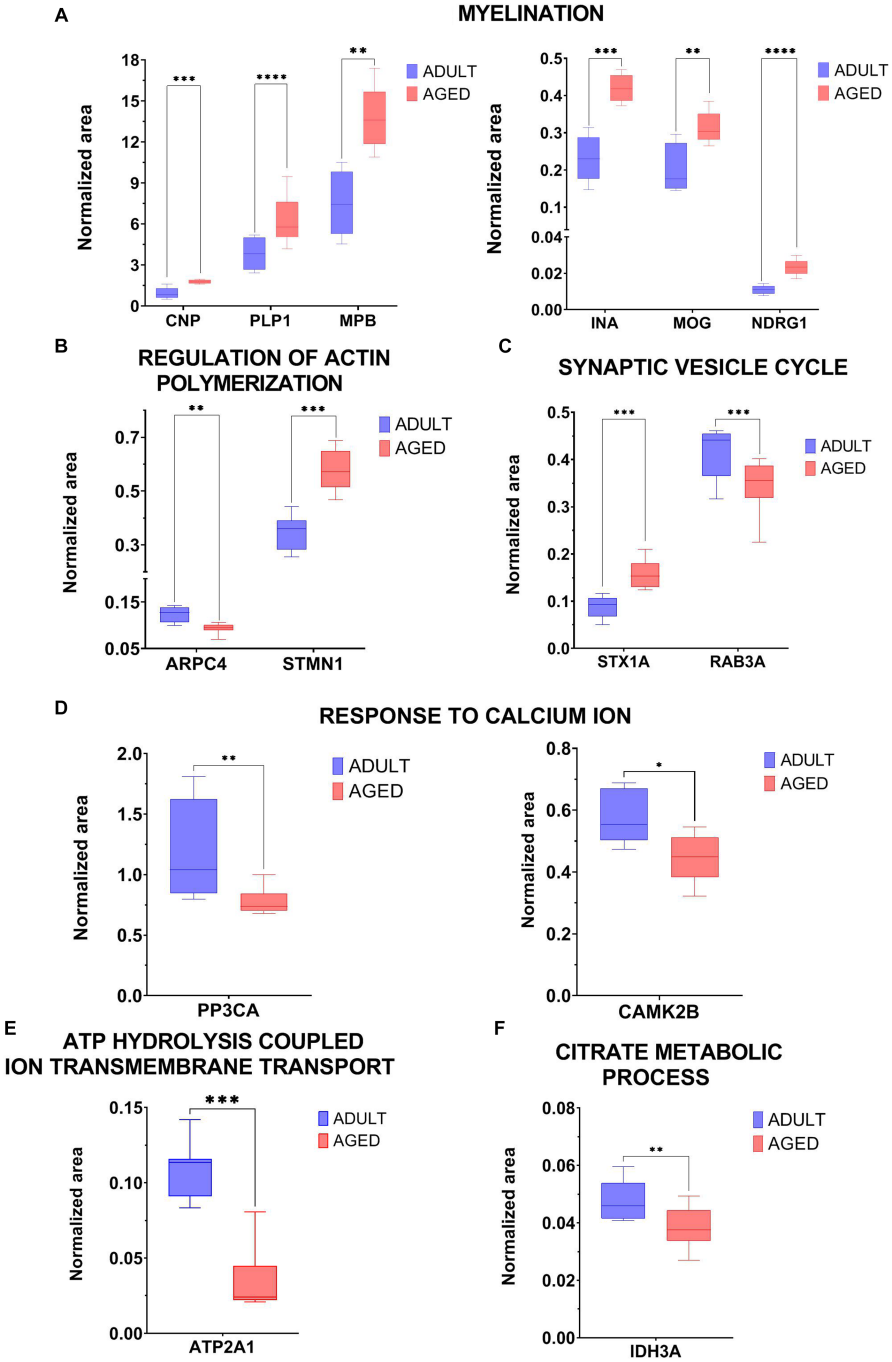
Graphical representation of MRM experiments. The normalized area of selected proteins measured in aged vs. adult samples were reported in two-dimensional graphs for specific proteins selected within the following biological processes: Myelination (CNP, PLP1, MPB, INA, MOG, NDRG1, panel **A**), regulation of actin polymerization (ARPC4, STMN1, panel **B**), synaptic vesicle cycle (STX1A, RAB3A, panel **C**), response to calcium ion (PP3Ca, CAMK2b, panel **D**), ATP hydrolysis coupled cation transmembrane transport (ATP2A1, panel **E**), citrate metabolic process (IDH3A, panel **F**). Statistical significance of protein expression calculated as aged/adult with a *value of *p* < 0.05, **value of *p* < 0.01, ***value of *p* < 0.001 and ****value of *p* < 0.0001.

The analytical data were processed by GraphPad Prism 9 and the intensity of the normalized area for each analyzed protein is reported in graph in Figure 3.

## 4. Discussion

In the present work, we sought to investigate the potential impact of aging upon proteomic profiling in the hippocampus of grazing cattle. The choice of comparing adult rather than young female subjects was due to both sexual maturity and productive phases of Podolica cattle breed, in order to study animals during the lactation persistency. We identified 112 proteins, associated to different molecular processes, including myelination, regulation of actin polymerization, synaptic vesicle cycle and calcium homeostasis. Hippocampus is a subcortical region of the limbic system, involved in the context-dependent cognition, as well as regulation of emotional behaviors. An overall age-dependent cognitive decline was observed in both humans and animals (Leal and Yassa, 2015; Bettio et al., 2017). Studies performed on animal models accordingly documented that old female rats showed cognitive dysfunctions and, hippocampal atrophy (Driscoll et al., 2006; Cole and Franke, 2017; Cole et al., 2019), thus highlighting a valuable role for the such a brain region in age-related impairments across sp. (Du et al., 2006). Cognitive deterioration during aging may be associated to physiological changes in molecular and neuronal pathways throughout life that, in turn, affect synaptic plasticity and connectivity within the hippocampus (Shivarama Shetty and Sajikumar, 2017).

### 4.1. Synaptic plasticity

Synaptic plasticity is regarded as structural and functional changes of both dendritic spines and their calcium-dependent postsynaptic density, after learning process (Bettio et al., 2017; Shivarama Shetty and Sajikumar, 2017). We documented a significant lower density of the calcium/calmodulin-regulated protein phosphatase, CAMK2B, which is involved in dendritic spine and synapse formation, and reorganization of the actin cytoskeleton (Sanabria et al., 2009) during plasticity, by binding and bundling actin filaments. This structural function is required to promote dendritic spine and synapse formation, and maintain synaptic plasticity, which enables Long Term Potentiation (LTP) onset and hippocampus-dependent learning (Borgesius et al., 2011; Lisman et al., 2012). In developing hippocampal neurons NMDARs promote arborization of the dendritic tree and in mature neurons, as well as dendritic remodeling and migration.

### 4.2. Synaptic vesicle cycle

The soluble N-ethylmaleimide-sensitive factor attachment protein receptor (SNARE) complex is involved in several processes within the brain, such as neurotransmitter release, autophagy and synaptic plasticity, and it can be impaired following physiologic aging. The core SNARE complex proteins SNAP25, VAMP2 and syntaxin 1 (STX) mediate synaptic vesicle fusion, exocytosis, and secretion of both neuropeptides and neurotrophins (Sudhof and Rothman, 2009), whereas the GTP-binding protein rabphilin 3A (Rab3A) acts as a negative modulator of vesicle fusion at presynaptic sites (Leenders et al., 2001). Similarly, STX1A localized at the synaptic plasma membrane within neurons, plays a key role in the modulation of several plasma membrane-bound monoamine transporters, such as serotonin, dopamine, and norepinephrine (Quick, 2003; Sung et al., 2003; Carvelli et al., 2008). In a previous work, STX1A has been associated to Aβ oligomers accumulation, which directly inhibit SNARE-mediated exocytosis in APP/PS1 TG mice, and considered the main pathogenic culprits for AD-related deficits in learning, memory and synaptic plasticity (Bornemann and Staufenbiel, 2000; William et al., 2012; Yang et al., 2015). Moreover, morphological and biochemical studies from Paciello and colleagues revealed a higher intraneuronal lipofuscin and APP immune-positive accumulation in both dentate gyrus and hippocampus of aged bovine (De Biase et al., 2017). Data from our exploratory study revealed that both STX1A and Rab3A proteins were differentially expressed in the hippocampus of old samples, allowing us to hypothesize a potential correlation between aging, Aβ aggregates and cognitive decline in cattle. By our unbiased approach, we did not identify proteins associated with Aβ metabolism and/or its clearance, such as BACE1, Nicastrin, IDE, APP. Differently, Tau is present among the statistically significant proteins, although with a FC value = 1.28, at the limit of biological relevance (Supplementary Table S1). On the other hand, the analysis of the variations of its phosphorylation level confirms its over-phosphorylation at least in correspondence of Ser209 (FC = 3.84) and Thr410 (FC = 5.19). These findings suggest that Tau hyperphosphorylation, a well-known hallmark of AD, might be also indicated as an age-related target in healthy subjects, as already reported (Noble et al., 2013; Carlyle et al., 2014).

### 4.3. Myelination

The changes of some myelin-related proteins in the hippocampus of old samples, prompt us to postulate that the aged-dependent accumulation of such an electrical and lipid-based insulator occurred, most likely due to its improper turnover, might be linked to the cognitive decline (Bennett and Madden, 2014; Wang F. et al., 2020; Murray et al., 2023). Our data were further confirmed by the higher density of the N-Myc downstream regulated 1 protein (NDRG1) in the aged samples. This iron-regulated growth and metastasis suppressor is known to be enriched in the cytoplasm of myelinating cells and, when mutated, associated to the onset of peripheral and myelin-based neuropathies in humans, such as Charcot Marie Tooth (Berger et al., 2002). Lack of NDRG1 was associated to a high rate of demyelination rate in knockout mice treated with the cuprizone diet, when compared to control animals, thus suggesting that NDRG1 might play a trophic role for peripheral nerve by trafficking and transport within the cytoplasm of Schwann cells (Marechal et al., 2022). In this line, previous immunofluorescence and molecular studies showed a significant age-dependent accumulation of myelin debris in the microglia cells of cortex, hippocampus and corpus callosum of Plp-DsRed transgenic old mice, that could be enhanced by the microglial phagocytic activity (Theriault and Rivest, 2016; Hill et al., 2018). This apparent discrepancy between myelination process and physiologic hippocampal aging might be related to the long-term stability of myelin-related proteins, that eventually leads to a progressive myelin breakdown and cognitive decline as well (Readhead et al., 1994). Further studies, aimed at better disclosing the functional role of the physiological age-dependent myelin degeneration within the hippocampus of bovine are mandatory.

### 4.4. Regulation of actin polymerization

Among a variety of molecular and cellular pathways involved in the brain physiology, dynamics of actin filaments and microtubule assembly have been regarded as key players of brain development, cell division and proliferation, as well as neurite outgrowth, axon migration and synaptic plasticity (Kim et al., 2022). Accordingly, proteomic and behavioral studies in rats documented age-dependent changes of the hippocampal actin-related protein 3 (ARP3) levels, associated to decay in long-term depression, and spatial learning and memory (Curmi et al., 1999; Ottis et al., 2013). The core subunit of the actin-related protein (ARP2/3) complex, ARPC4, which catalyzes the formation of F-actin networks, has been associated to neural migration and differentiation (Laboy Cintron et al., 2022). Moreover, the ubiquitous phosphoprotein Stathmin 1 (STMN1) was found expressed in brain regions associated to learning-dependent changes in microtubule stability; previous findings showed that genetically modified stathmin mice showed age-dependent alterations in the strength of synaptic responses, anxious hyperactivity and impaired hippocampal long-term potentiation (Uchida et al., 2014; Nguyen et al., 2019). Therefore, the altered expression of both ARPC4 and STMN1 proteins in the hippocampus of our old samples let us hypothesize and confirm that a cognitive impairment may also occur in bovine during the physiologic aging.

### 4.5. Mitochondria metabolism

Cytoscape analysis showed an age-dependent modulation of pathways underlying aerobic respiration and TCA-related processes, since a lower abundance of mitochondrial markers, including vesicular glutamate transporters, isocitrate and dehydrogenase subunits and succinate dehydrogenase was found in the old hippocampal samples. Given that mitochondria are regarded as both source and target of the reactive oxygen species (Grimm and Eckert, 2017), our data allow us to better understand the role of age-related mitochondrial dysfunctions during physiological aging in the hippocampus of dairy cattle, although they require to be further validated by means of LC–MS/MS analysis.

Alongside welfare and ethics, aging in farm animals is gathering a growing interest for society. Indeed, in recent years animal welfare is progressively shifting from the five freedoms as such, animals’ needs, affective states, and inter-individual differences, to the importance of having knowledge about the ability of acquiring, processing, and using information, in order to improve livestock management and practices, in particular during production. In this framework, according to Shettleworth’s theory, cognitive mechanisms can be better understood if physical and social cognition are taken into account, in order to evaluate the ability of farm animals to solve particular tasks (Shettleworth, 2001). Physical cognition in cattle is generally based on their ability to learn and be aware of the environment, allowing them to associate the location with quantity and quality of food found there, and improve the foraging patterns (Nawroth et al., 2019). Cognitive abilities can be adequately preserved if the animals are allowed to perceive and address their social and physical environment, thereby improving housing, management practices and production as well. In this respect, housing conditions (especially from intensive livestock) are often limited, regarding species-appropriate structure and behavior, thus causing the animals to develop stress and reduced welfare. Enabling them to be housed in the presence of cognitive enrichment and suitable environment, might turn into a functional improvement of positive states and wellbeing. Cattle used in the present study were all Podolica breed, female, with no parental bond. Animals came from several and different family-run extensive farms in Salerno province, in Southern Italy. Cattle had the freedom to wander outdoors, and the autonomy over the access to shelter, water consumption and diet selection mostly by grazing on large areas of pastures and natural resources. Moreover, the animals were held in farms that offered high welfare conditions, such as protection from predators, extreme changes of temperature and allowed them to form natural bonds and hierarchies and to be free from thirst, hunger, and fear. Therefore, their welfare was not negatively influenced by intense noises, human activities, inadequate husbandry management or poor air quality. Differential proteomic profiling underlying myelination, synaptic plasticity and calcium homeostasis processes identified in old Podolica cattle, are broadly consistent with pathway changes observed in other animal systems (Table 1).

**Table 1:** Some of the potential aging biomarkers identified in this study are summarized considering the biological process in which they are involved and the observed disorders in which they have been studied in correlation with aging in other organisms.

Biological process	Associated proteins	Organism	Associate disorders	Bibliography
Mitochondria metabolism	ATP2A1	Mouse	Neurological deficit; Myotonic dystrophy (DM) 1 and 2	Hasuike et al. (2022)
	ATP6V0A1	Mouse; Human	Neurodevelopmental disorders; neurological deficit	Aoto et al. (2021)
	IDH3A	Mouse	Retinal degeneration	Findlay et al. (2018)
	IDH3B	Mouse	Retinal degeneration	Findlay et al. (2018)
	IDH3G	Mouse	Retinal degeneration	Findlay et al. (2018)
	CAT	Mouse; Human	Age degenerative diseases; PD, AD	Nandi et al. (2019)
	FABP3	Mouse; Human	Synucleinopathies	Oizumi et al. (2021)
	APOD	Mouse; Human	AD, PD, stroke, multiple sclerosis, schizophrenia, meningoencephalitis	Rassart et al. (2020)
Myelination	PLP1	Mouse	Neurological deficit	Abdelwahab et al. (2023)
	MOG	Mouse	Neurological deficit	Gil et al. (2010)
	MBP	Mouse	Neurological deficit	Wang Y. et al. (2020)
	NDRG1	Mouse	Neurological deficit	Marechal et al. (2022)
	CNP	Mouse	Neurological deficit	Baburina et al. (2017)
	INA	Mouse; Human	Neurological deficit	Yuan and Nixon (2021)
Regulation of actin polymerization	ARPC3	Rat; Mouse	Synaptic dysfunction: poor learning and memory; behavioral abnormalities	Ottis et al. (2013)
	ARPC4	Mouse; Human	Microcephaly and speech delay	Cintron et al. (2021)
	STMN1	Mouse; Human	Aging	Li et al. (2016)
	ARPC2	Mouse; Human	General senescence, aging process	Haarer et al. (2023)
	CFL2	Mouse	Involvement in Alzheimer’s	Wang et al. (2007)
	CAPZA2	Mouse; Human	Neurodegeneration; Alzheimer’s disease	Suszyńska-Zajczyk et al. (2014)
Synaptic plasticity	CAMK2B	Mouse	Impairment in learning	Lisman et al. (2012)
	PP3CA	Mouse; Human	Deficit in brain and memory; traumatic brain injury	Wu et al. (2013)
	HPCA	Rat	Cognitive impairment	VanGuilder et al. (2011)
	TAU	Monkey	Aging	Carlyle et al. (2014)
Synaptic vesicle cycle	STX1A	Mouse	Neurological deficit	Fujiwara et al. (2016)
	RAB3A	Mouse	Accumulation of Aβ-peptides: Alzheimer’s disease (AD)	Szodorai et al. (2009)
	SYT1	Mouse; Human	Memory deterioration; AD	Kuzuya et al. (2016)
	SYNJ1	Mouse	Parkinson’s disease (PD)	Zhu et al. (2023)
	SLC25A11	Mouse	Neurodegenerative diseases associated with aging	McCormick and Promislow (2018)
	SLC25A22	Mouse	Neurological deficit; AxD and VWM disease	Hillen and Heine (2020)
	SLC17A7	Mouse; Macaques	Neuronal impairment linked to aging	Eghlidi et al. (2018)

Collectively, despite the lack of a cognitive correlate of protein changes observed in the hippocampus of old cattle, our preliminary data obtained from an unbiased approach pave the way for further investigations on a larger cohort of grazing animals, aiming at improving animal welfare and husbandry practices of dairy cattle from intensive livestock.

## Data availability statement

The datasets presented in this study can be found in online repositories. This data is available via ProteomeXchange: http://www.ebi.ac.uk/pride/archive/projects/PXD044404.

## Ethics statement

Ethical approval was not required for the studies involving animals in accordance with the local legislation and institutional requirements because Even though our research includes animal tissues, the study did not require consent or ethical approval according to European Directive 2010/63/EU because all sampling procedures from animals were performed during post-mortem inspection. However, the animals were slaughtered rigorously in line with European regulations (CE no: 1099/2009 of 24 September 2009) that assure the protection and welfare of animals at the time of killing. The owner of the abattoir and the veterinary inspector responsible for the sanitary surveillance granted permission to collect the samples. Written informed consent was obtained from the owners for the participation of their animals in this study.

## Author contributions

MM: Conceptualization, Resources, Supervision, Writing – original draft, Writing – review & editing. FC: Investigation, Writing – original draft. LC: Methodology, Validation, Investigation, Writing – review & editing. LS: Software, Writing – original draft. MG: Methodology, Validation, Writing – review & editing. II: Data curation, Formal analysis, Software, Writing – review & editing. CG: Project administration, Visualization, Writing – review & editing. DB: Resources, Visualization, Writing – review & editing. EN: Resources, Visualization, Writing – review & editing. OP: Resources, Visualization, Writing – review & editing. LA: Project administration, Visualization, Writing – review & editing. Dd’A: Conceptualization, Funding acquisition, Project administration, Writing – review & editing. FN: Conceptualization, Project administration, Writing – original draft, Writing – review & editing.
